# New Means of Extracting Opium, &c. from the Stomach

**Published:** 1822-11

**Authors:** Edward Jukes

**Affiliations:** Surgeon, Westminster.


ART. II.—*New Means of extracting Opium, &c. from the Stomach.* By EDWARD JUKES, Surgeon, Westminster.

A FEW days after the unfortunate decease of the late Primate of Ireland, I published a plan for removing, by mechanical agency, poison from the stomach, in those cases where this organ had become insensible to the stimulus of emetic substances. My remarks seem to have excited pretty generally the consideration of professional men, as several papers have since appeared in the most recent medical publications on this subject.

My thoughts were so simple, and the apparatus which I constructed is possessed of so little ingenuity, that I could never have considered them as deserving of praise; much less could I have expected my notions would have been deemed worthy of piracy. I beg, therefore, to present to the readers of the London Medical and Physical Journal an epitome of my suggestions and experiments, as detailed in several past Numbers of a periodical publication of the present year.

"I offer the following suggestions, then, not as perfected and ready for adoption, but as ideas hastily collected, and submitted for the improvement which ingenious minds will not fail of producing.

"I submit, therefore, that if the vitality of the stomach (in cases of poisoning by opium,) has been so far reduced by the narcotic drug, that the organ is insusceptible to the stimuli of emetic medicines, or is so torpid and enfeebled that it is incapable of assuming the actions necessary to vomiting, then it is incumbent on the surgeon to resort to *mechanical* means for dislodging the poison from the stomach, which may be effected by an apparatus of the following nature:

"1st. A hollow flexible tube (of elastic gum), about twenty-five inches in length, and half an inch in diameter, having three equidistant longitudinal grooves, of an inch and a half in length, around its extremity. Within each groove three holes, which perforate the tube, of one-eighth of an inch in diameter, (though, perhaps, it might be as well to make them oblong, like the eye of a catheter,) and rather less than half an inch asunder. The opposite extremity of the tube to be fitted with a small screw, to be adapted to the other part of the apparatus.

2

"2d. An elastic bottle, capable of holding a quart in measure, armed with a short pipe and stop-cock, (similar to an hydrocele bottle,) the extremity of which may be screwed into the end of the tube above mentioned.

"With such a simple apparatus, I conceive the stomach might be emptied, when emetics have been tried in vain; and I should employ it in the following manner:

"The tube having been carefully passed down to the greater curvature of the stomach, *the elastic* bottle being filled with water of the temperature of 150° of Fahrenheit's thermometer, is to be screwed into it; and, the stop-cock being opened, the contents of the bottle is to be gently urged through the tube into the stomach by the surgeon, or an assistant, compressing the bottle between his hands. By removing this pressure, the bottle, by its elasticity, recovers its original form and dimensions, and consequently performs the office of an exhausting pump, by which means the fluid is again drawn back into it.

*Remarks.*—I would use water of the high degree of temperature that I have mentioned, with the view of obtaining some effects from its stimulus. We know, from experimental observation, that there is no stimulant capable of rousing the suspended functions of animal bodies so certainly as the matter of heat; and to prove this it is only necessary to take some one of the hybernating animals, during its torpidity, and submit it to the action of galvanism and to immersion in warm water: the superior influence of the latter over the former, in restoring the external phenomena of life, is soon rendered apparent."

"But it is not from temperature that I expect the greatest benefit to arise by the injection of water, but from dilution. It must be remembered that the active elements of the poison are contained in a very small bulk; and, even should a wine-glassful of laudanum have been swallowed, such a quantity may easily be enveloped in the folds of the stomach, and thus escape the action of the apparatus: distending the stomach, then, with water will equally diffuse the poison. Besides which, if solid food should be contained in the stomach at the time of taking the laudanum, it may so far *absorb* it as to render the removal of the remaining liquid contents of the stomach of no avail; but the injected warm water pervading every recess of the stomach, and permeating its solid contents, would most probably wash away the whole of the poison contained within it. The operator should not be content with once injecting and evacuating the stomach; he should continue to throw in a fresh quantity of water, as long as the fluid abstracted betrayed any signs of being impregnated with the poison.

"It is proper to remark that it will not be necessary to inject the stomach, except in those instances where the patient has become comatose; if he remain sensible, he should be directed to drink copiously of hot water, previously to the introduction of the tube into the stomach.

"It will, perhaps, be asked what method I devise for recovering the patient from the effects of the poison already produced? I reply, that, had I at this time leisure to enter into this part of the subject, it would be foreign to my purpose; my intention, at present, being simply to propose some means for evacuating the poison by mechanical agency, when the vital energy itself is incapable of doing so.

"I have advised the holes in the extremity of the tube to be made within the channel of several grooves, and for this reason, that, without such a precaution, as soon as the suction is made, the mucous membrane of the stomach, if it should happen to be in contact with the instrument, might be drawn into the openings, and thus prevent the discharge of its contents, as well, perhaps, as sustain some injury itself: it is obvious that the ridges between the grooves will tend materially in preventing such an occurrence.

"It has occurred to me that, even in those desperate circumstances where the excitability of the stomach is reduced so low that emetics will not act, this organ may, by mechanical means, be brought into such a condition as will render it susceptible to this class of medicines; —that is, that such a temporary influx of the nervous principle may be elicited to it, by mechanical irritation, as will renew sufficient excitability of the stomach to render it sensible to the stimulus of emetic substances, and give its muscular structure sufficient energy to enable the organ to excite the associated motions of sympathetic parts, and to fulfil the actions of vomiting. To ascertain this, I am about trying the effect of introducing the dolichos, in a *dry state*, into the stomach. Whether the viscidity of the mucous fluid of the stomach will protect the inner surface from the mechanical irritation of this article, I am not aware; nor am I at all acquainted with the effect which it might have upon the gullet, fauces, and mouth, if it were thrown up by vomiting. A physician of my acquaintance has, indeed, informed me that he has swallowed the hairs of dolichos, mixed with water, without experiencing the slightest inconvenience; but experiment alone can determine it. I shall administer, first, an emetic; and, having waited some minutes, I intend to pass the dolichos into the stomach, through a hollow tube, open at both extremities. Should this fail, I shall endeavour to excite the stomach by passing a fasciculus of bristles, secured to a flexible stillet, through the tube, and gently irritating the stomach with it.

"Having thrown these crude notions together, I leave them for the consideration and improvement of those as anxious as myself. They will, no doubt, be found extremely defective, because theoretical; but, should they only prove the humble means of eliciting an attention to the subject, which shall eventually lead to practical utility, I shall consider them not altogether useless."

"*Westminster; May 29th*, 1822."

During the month of June, having but little leisure, I made but slight progress in the prosecution of my original views; the following account of my experiments was published on the 1st of July:

"*Experiment 1st.*—An elastic gum tube, fitted to a pint bottle, (of a similar material,) was passed into the stomach of a moderate-sized terrier dog, and a pint of warm water thrown into it, by compressing the vessel. Upon removing the pressure, the bottle, by its elasticity, regained its usual dimensions, by which a vacuum being formed, the liquid contents of the stomach rushed through the tube, and instantly re-filled the bottle.

" *Experiment 2d.*—Two ounces of laudanum was swallowed by the same animal, and, having remained a few minutes, a pint of warm water was injected into the stomach, by the before-mentioned apparatus. The liquid returned, highly tinged and flavoured with opium. Another pint of water was now thrown in, which returned but slightly impregnated with it; and a third quantity, being introduced and withdrawn, showed no signs of any thing foreign, except here and there a floating flake or two of the mucus of the stomach. The dog, being loosed, betrayed no symptoms of uneasiness, and soon afterwards made a hearty meal; no effects from the narcotic following.

" *Experiment 3d.*—Two drachms of laudanum were forced into the throat of a large spaniel, and in a few minutes afterwards a pint of warm water, injected through the tube, passed into the stomach. The water returned, mixed with a good deal of the chymous contents of the stomach, smelling strongly of the laudanum. A second pint of water was thrown in, which returned mixed with the same pulpy, demi-gelatinous matters as before, and impregnated, though less sensibly than the former, with opium. A third pint of water returned only a little turbid, and the faint peculiar smell of opium was scarcely to be recognized. A fourth portion, when withdrawn, was hardly at all changed, except in losing its transparency, and neither the taste nor smell evinced the presence of opium. *The animal was not subsequently the least affected.*

" *Remarks.*—By the first experiment, it was established that the stomach of the animal might be emptied by the apparatus used.

" The second experiment proved that the laudanum contained in the stomach being diluted by, and withdrawn in, the injected liquid, no harm resulted to the dog from the administration of the poison.

" My object in the third experiment was to ascertain if there was any liability of failing in diluting a *smaller* quantity of laudanum in the stomach of a *larger* dog; and the result was, that the stomach of the animal, containing some quantity of food, required the more frequent application of the apparatus to withdraw its contents; but, at the same time, I think it probable that, had this organ been empty, a larger quantity of fluid would have been requisite (to distend the stomach so sufficiently that none of the laudanum should have escaped, by being concealed in its plicæ,) than was used in the former experiments.

" Not having obtained a perfect apparatus for conducting my experiments, beyond the foregoing, in a satisfactory manner, I shall not at this time enter into a farther detail. I expect, in a few days, to be furnished by Mr. Gill, surgical instrument maker, of Bedford-row, with such an improved apparatus as shall ensure the full success of the proposed practice, when I shall resume my experiments and their report."

" *Westminster; June 26th,* 1822."

In August I satisfactorily proved, by direct experiments upon myself and others, that my apparatus was fully competent to the purpose of drawing off the contents of the stomach safely and effectively, as appears by the following recital of the 1st of September:

"On the 12th instant, I swallowed a drachm of laudanum, undiluted, and immediately afterwards a pint of tepid water. Having my apparatus ready, I passed the flexible tube into the stomach, and drew off *rather more than a pint* of fluid, mixed a little with the previous contents of the stomach, and smelling strongly of opium. Being satisfied, from the perfection of the instrument, that the stomach was emptied, I desisted from any farther experiments, and sat down to wait for any sensations that might be produced; *but I experienced none*, and after a short time ate my dinner with appetite.

"*August* 14*th.*—This day, at noon, when I concluded the stomach was empty, I swallowed *two* drachms of undiluted laudanum, and immediately afterwards a pint of warm water. This was instantly drawn off, as in the former experiment, and occasioned me no subsequent sensations.

"15*th.*—I drank, this morning, half an ounce of laudanum; and having as quickly as possible diluted it, by drinking a pint of warm water, the tube was passed, and the liquid withdrawn. In this experiment, as in the two preceding, I experienced no effects from the opium.

"16*th.*—At two o'clock P.M. I swallowed *ten* drachms of laudanum, in the presence of my friend Mr. James Scott; and, having immediately afterwards drank a quart of tepid water, Mr. Scott passed the tube into my stomach, and drew off a pint of fluid, strongly impregnated with opium; and having emptied the elastic bottle, and again applied it, drew off another pint of a similar liquid.

"The bottle was now filled with warm water, which, being forced through the tube into the stomach, returned, flavoured *slightly* with opium. Another pint of warm water was now, in the same manner, injected into the stomach, and, when withdrawn, exhibited to the taste and smell but the faintest trace of the medicine. Another pint of water was, however, thrown in, and withdrawn; when Mr. Scott feeling perfectly satisfied of the entire removal of the laudanum, the experiment, which had occupied about ten minutes, was discontinued.

"I began now, however, to experience sensations of nausea and giddiness, which, though they might perhaps have been occasioned by the absorption of a portion of laudanum, I am myself inclined to attribute to the irritation of the fauces and stomach, produced by the instrument, and the large quantities of warm water. The nausea increasing, and a disposition to sleep supervening, I threw myself into a recumbent position, and soon lost the disagreeable feelings in a sleep which continued profoundly for three hours. I had slight head-ach on awaking; my tongue was loosely covered with an orange fur; and, though my usual dinner-hour had passed, I had no disposition to eat. I now drank several cups of strong coffee, and in a very short time was restored to my usual state of health and feelings; and having taken, during the evening, occasional draughts of lemonade, the desire for food returned, and I ate some supper, as though my stomach had not been disturbed.

"Having ascertained, by experiments on my person, that laudanum might be safely introduced, even in very considerable quantities, into the stomach, I took the earliest opportunities of repeating them upon others; and, in three instances in which I have subsequently administered an ounce of laudanum, (one of which was a female,) the poison was extracted with the usual apparatus, without the slightest symptoms of uneasiness remaining to the patient."

The apparatus that I now use consists of an elastic gum hollow tube, a quarter of an inch in diameter, and two and a half feet in length, having affixed at one extremity a small globe of ivory, *with several perforations;* the other extremity is adapted, either by screw or by plug, (the latter is preferable,) to an elastic *bottle*, of sufficient size to contain at least a quart of liquid, having a stop-cock fitted to it, (similar to an hydrocele bottle,) or, instead of the *bottle*, a pewter syringe of an equal capacity, adapted to the flexible tube in the same manner: (the operation by the syringe is performed more quickly, and may perhaps, therefore, by some be preferred.) In cases where surgeons have neither bottle nor syringe, the tube alone might be made to answer the purpose, by the operator's applying his mouth to its extremity, and thereby instituting the office of a syphon.

In the use of my instrument, I place the patient on the *left side*,* and having passed the tube, either by the mouth or the nostril, into the stomach, I inject from the bottle a quart of water, heated to 150° of Fahrenheit, and withdraw it in the manner formerly noticed.

*Great Peter-street, Westminster; Sept.* 13, 1822.

* For this suggestion I thank Mr. Bush, of Frome.

